# Platelet Rich Plasma Therapy in Non-insertional Achilles Tendinopathy: The Efficacy is Reduced in 60-years Old People Compared to Young and Middle-Age Individuals

**DOI:** 10.3389/fnagi.2015.00228

**Published:** 2015-12-10

**Authors:** Vincenzo Salini, Daniele Vanni, Andrea Pantalone, Michele Abate

**Affiliations:** Orthopaedic and Traumatalogical Clinic, Department of Medicine and Science of Aging, Università degli Studi “G. d’Annunzio” Chieti-PescaraChieti, Italy

**Keywords:** Achilles tendinopathy, aging, platelet rich plasma, young, ultrasonography

## Abstract

**Background:** Platelet Rich Plasma (PRP) has shown positive and long-lasting effects in patients with tendinopathies. However, information about age-related differences in the clinical outcome is limited. Aim of this retrospective study was to compare the efficacy of PRP therapy in young and elderly subjects suffering for Achilles tendinopathy.

**Materials and method**: Patients with recalcitrant non-insertional Achilles tendinopathy were enrolled. Clinical (VISA-A) and instrumental (ultrasonography) data were collected at baseline and after 1, 3, 6, and 12 months. PRP injections (once a week for 3 weeks) were performed in sterile conditions and under ultrasound (US) control.

**Results:** Forty-four subjects (29 young: mean age 39.5 ± 6.9; 15 elderly: mean age 61.5 ± 5.3) were retrospectively evaluated. At baseline, no significant differences were observed in the clinical and US parameters. Throughout the whole length of the study, a significant increase of VISA-A score was seen in both groups (from 50.3 ± 8.8 to 76.1 ± 6.6 in the young group, and from 48.7 ± 7.6 to 61.1 ± 9.4 in the elderly group); however, the infra-groups comparison showed better results in young patients, compared to the aged counterpart.

**Conclusion:** Our results show that PRP is less effective in aged people. This finding can be ascribed to several biochemical and biomechanical differences documented in tendons of young and elderly subjects (reduced number and functionality of tenocytes and tenoblasts), which becomes more evident in the long-term tissue healing. However, prospective trials, using different PRP preparations and enrolling a larger number of subjects, are needed to draw more sound and definitive conclusions.

## Introduction

In the last decade Platelet Rich Plasma (PRP) has been extensively used in the treatment of tendinopathies. Many trials have been performed on different tendons, and several PRP preparations and treatment schedules have been proposed. These studies, broadly speaking, have shown positive and long-lasting effects on symptoms and function in a large percentage of cases (Andia and Abate, 2012; Andia and Maffulli, 2013; Andia et al., 2014). A superiority of PRP in comparison to placebo or other usual treatments (e.g., eccentric training, physical therapies, steroid injections) has been observed by some authors, but not by others (Andia and Abate, 2012; Andia and Maffulli, 2013; Kearney et al., 2013; Andia et al., 2014).

Despite the large amount of data gathered in these experiments, information about possible difference in efficacy age-related is limited. In particular, at our knowledge, only in few papers this topic has been addressed with inconclusive results, evaluating different factors (age, sex, BMI, duration of symptoms, severity of degeneration, and others), which can theoretically influence the clinical outcomes (Ferrero et al., 2012; Boesen et al., 2014; Filardo et al., 2014).

This is an important point, because the prevalence of tendinopathies nowadays is increasing in aged subjects, who practice frequently sport activities, both for leisure and for counteracting with exercise metabolic diseases (i.e., diabetes and obesity) (Dallaudière et al., 2013). In this framework, it must be considered that aging itself may affect biochemical and biomechanical properties of tendons, so favoring the onset of tendon damage.

Non-insertional Achilles tendinopathy is a very common disease, mainly in sport-active population. Aim of this retrospective study was to compare the efficacy of PRP therapy in young and elderly subjects, addressed to our unit for recalcitrant non-insertional Achilles tendinopathy.

## Materials and Methods

Patients suffering from recalcitrant non-insertional Achilles tendinopathy treated beforehand in our Unit with PRP were retrospectively evaluated. Subjects who failed to respond to conservative treatments [e.g., eccentric training, laser, Extra-corporeal Shock Wawe, ultrasound (US), and steroid], with history of exercise-associated pain, pain or tenderness on palpation more than 3 months, and US features of chronic non-insertional damage in the Achilles’ tendon were included. Exclusion criteria were: insertional Achilles tendinopathy, symptom duration <3 months, platelet values <150.000/mm3, Hemoglobin values < 11 g/dl, bleeding disorders, current use of anticoagulants or antiaggregants, hematological and rheumatic pathologies, severe systemic diseases (renal, hepatic, cardiac, infections, endocrinopathies, malignancies), immunodepression, and pregnancy (**Table 1**).

**Table 1:** Inclusion and exclusion criteria.

Inclusion criteria	Exclusion criteria
Non-insertional Achilles tendinopathy	Insertional Achilles tendinopathy
Symptom duration >3 months	Symptom duration <3 months
No response to conservative treatments	Platelet values < 150.000/mm3
	Haemoglobin values < 11 g/dl
	Bleeding disorders
Ultrasound features of tendon damage	Use of anticoagulants/antiaggregants
	Rheumatic pathologies
	Severe systemic diseases
	Immunodepression
	Pregnancy


At baseline, in all subjects, demographic (age and sex) and anthropometric measures (height, weight, and BMI) were registered. Clinical (symptoms duration, weekly non-steroidal anti-inflammatory drugs consumption, associated diseases, sport practice) and functional data [Victorian Institute of Sports Assessment- Achilles questionnaire (VISA-A), adapted to the Italian language] were also collected. The VISA-A provides a subjective functional evaluation of Achilles tendon, and consists in eight questions which measure the domains of pain and function in daily living and sporting activity (Abate et al., 2013). Results range from 0 to 100, where 100 represent the perfect score.

Ultrasound evaluation was performed by the same well-trained operator (AM) using a high-resolution, multi-frequency (6–15 MHz) linear array transducer (*ProSound Alpha 10, Aloka, Japan*). Longitudinal and transverse scans were performed according to standard protocols (Maffulli et al., 2008) with the patient lying prone, with the feet hanging over the edge of the table at 90° of flexion. Loss of the normal fibrillar pattern, and/or irregularity of the tendon margins, and focal hypo-hyperechoic areas in the musculo-tendinous junction and/or in the mid-portion were considered as degenerative abnormalities. On the basis of these features tendons were stratified for severity as “mild” (one area of disorganized echotexture), “moderate” (some areas of disorganized echotexture), and “severe” (disorganized echotexture and diffuse hypo- or hyperechoic areas and/or calcifications). The presence of neovascularization, estimated by means of Color Doppler, was graded as (0), (1+), (2+), (3+), (4+), according to the appearance of vessels inside the tendon (Abate et al., 2013). To avoid artifacts, sensitivity was optimized for low flow, and color gain was set just below the noise level.

Platelet Rich Plasma was prepared using the Regen Lab A-PRP Kit. In detail, 8 ml of autologous blood was harvested from the cubital vein and collected into a tube containing a citrate anti-coagulant in addition to the thixotropic cell-separation gel. Then, the tube was carefully turn upside down several times (×5) to homogenize the blood with the anti-coagulant. After centrifugation [single spin, Force (RCF): 1500 *g*, 3400 rpm for 5 min], the blood was fractionated, with the red blood cells trapped under the gel, and the cellular sediment, including the platelets, settled on the surface of the gel. Therefore, by gently inverting the tube several time, the sediment was resuspended in the plasma supernatant, and PRP (4–5 ml, 1,6x native platelet concentration, >80% platelet recovery, no leukocytes, red blood cell remnant <0.3%) was obtained. PRP was then collected into a 10 ml luer-lock syringe and ready for use.

After sterile dressing and under US control, small autologous PRP depots were left at several sites into the degenerate tendon areas, using a 21 Gage needle. PRP was placed at the site of most damaged areas, for a total amount of 4–5 ml. No regional anesthetic was used. A total of three injections (once a week) was performed. After the second injection, a rehabilitation program, based on eccentric training and stretching, was recommended daily (3 sets × 15 repetitions) at least for 3 months, during which a gradual return to sport activities was encouraged. After each injection, the patients were kept under observation for approximately 30 min (monitoring early side effects) and then discharged from the Unit. At home, patients were asked to restrict the use of the leg for at least 24 h; rest, ice packs, and acetaminophen (non-steroidal anti-inflammatory drugs were forbidden) were allowed. Moreover, patients were asked to register possible adverse events (pain, swelling, heat, functional limitations) and acetaminophen consumption during the following days after the injection.

Functional and instrumental evaluations were repeated after 1, 3, 6, and 12 months, and patients satisfaction was registered by means of five-points Likert Scale (Not at all satisfied; Slightly satisfied; Somewhat satisfied; Very satisfied; Extremely satisfied).

### Statistical Analysis

According to age, patients were divided in two different cohorts (Young: <55 years old; Elderly: >55 years old. This partition was arbitrary done because a division universally accepted is not present). Demographic, US and clinical data, before and after treatment, were therefore compared.

Data are reported as mean ± standard deviation for continuous variables, whereas categorical and dichotomous variables are reported as frequencies and percentage. The significance level was determined at *p* < 0.05. The two-sample Student’s *t*-test was used to compare continuous variables, when the distribution of data was normal; the Wilcoxon’s rank sum test was used otherwise. The χ^2^ test was used to evaluate associations between categorical data. All analyses were done using SAS statistical software, release 8.1.

## Results

Forty-four subjects met inclusion criteria. Demographic and clinical data of enrolled patients are reported in **Table 2**, which shows that, apart of age, and the presence of three cases of diabetes (well-controlled, self-report) in the elderly group, no differences were observed for other collected parameters. Neither at the instrumental examination, significant difference in the US degeneration and in the neovascularization score was present (**Table 3**).

**Table 2:** Patients characteristics at baseline.

	Young	Elderly	*p*
Number	29	15	–
Tendons treated	36^∗^	18^∗∗^	–
Male:Female	19:10	13:2	–
Mean age	39.5 ± 6.9	61.5 ± 5.3	0.000
BMI	24.3 ± 1.8	25.9 ± 1.9	0.07
Symptoms duration (weeks)	28 ± 8.4	32.4 ± 6.5	0.1
Sport activities	20/29 (68.9%)	7/15 (46.6%)	0.14
Diabetes	–	3/15 (%)^∧^	0.01
NSAIDs consumption	5/29 (17.2%)	3/15 (20%)	0.8
VISA-A	50.3 ± 8.8	48.7 ± 7.6	0.5


*^∗^7 and ^∗∗^3 bilateral. ^∧ ^Well-controlled (self-report).*

**Table 3:** Ultrasound (US) findings at baseline in young and elderly patients.

	Young	Elderly	*p*
**US degeneration**			
Mild	10 (27.7%)	5 (27.7%)	0.7
Moderate	19 (52.7%)	9 (50%)	0.9
Severe	7 (19.4%)	4 (11.1%)	0.9
**Neovessels**			
Absent	6 (36.6%)	5 (27.7%)	0.5
Present	30 (83.3%)	13 (72.2%)	0.5
(1+)	8 (26.6%)	7 (53.8%)	0.1
(2+)	17 (56.6%)	4 (30.7%)	0.2
(3+)	3 (10%)	1 (7.6%)	0.7
(4+)	2 (6.6%)	1 (7.6%)	0.5


No complications related to the injections or severe adverse events were observed during the treatment and follow-up period. Four young patients (2 at 3 months and 6 months, respectively) and three elderly subjects (2 at 3 and 1 at 6 months, respectively) were lost at follow-up.

The variations of VISA-A score at different follow-up times in the remaining patients of both groups are shown in **Table 4**; an increase of VISA-A score is observed both in young and elderly subjects throughout the whole length of the study, although with different levels of significance (higher in the young).

**Table 4:** Victorian Institute of Sports Assessment- Achilles questionnaire (VISA-A) score during follow-up in young and elderly patients (intra-group comparison).

	VISA-A			
	
	Young	*p*	Elderly	*p*
Baseline	50.3 ± 8.8		48.7 ± 7.6	
1 month	56.8 ± 9.7	0.01	54.4 ± 7.1	0.04
3 months	68.7 ± 8	0.000	56.6 ± 4.9	0.002
6 months	72.4 ± 7.9	0.000	59.3 ± 8	0.000
12 months	76.1 ± 6.6	0.000	61.1 ± 9.4	0.000


**FIGURE 1:**
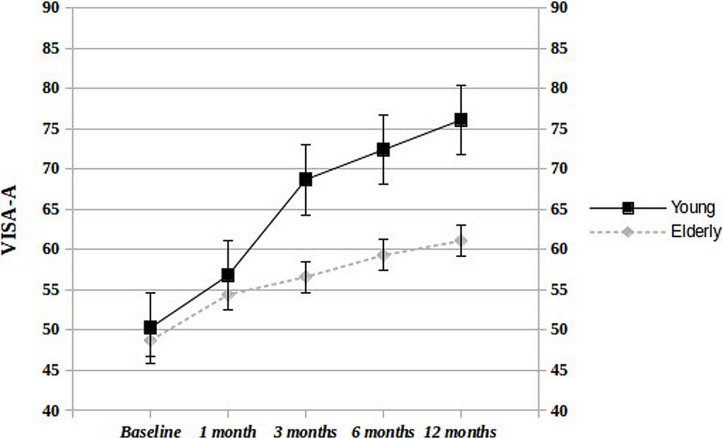
**Infra-group comparison at different follow-up times.** Significant differences in VISA-A score were observed at 3, 6, and 12 months (*p* < 0.000) but not at 1 month (*p* < 0.3).

The infra-groups comparison shows that values increase more steadily and consistently in young patients, compared to the aged counterpart (**Figure 1**). At 12 months, 18/25 (72%) young and 6/12 (50%) elderly patients were very/extremely satisfied from the treatment. The US evaluation at 12 months, compared to baseline, did not show any significant variation in both groups.

## Discussion

The results of this study show that PRP treatment provides satisfactory results in young subjects with Achilles recalcitrant non-insertional tendinopathy reducing pain and improving function. These findings are in agreement with previous literature data in patients suffering from Achilles, patellar, and elbow tendinopathies (Andia and Abate, 2012; Andia and Maffulli, 2013; Andia et al., 2014). It is current opinion that the therapeutic activity of PRP is mainly due to the release of several growth factors (GFs), which can act on different aspects of tendon repair, including angiogenesis, chemotaxis, and cell proliferation by activating intracellular signal-transduction pathways (Anitua et al., 2006; de Mos et al., 2008; Abate et al., 2015).

In the short term (1–3 months), GFs can directly stimulate tenocytes to produce extracellular matrix, and promote neofibrils formation and remodeling. Indeed, it is well known that the Growth Hormone/Insulin-like GF-1 axis plays a central role in the regulation of human collagen turnover in musculo-tendinous tissue (Doessing et al., 2010; Andia and Abate, 2013). Insulin-like GF-1 stimulates collagen formation (Abrahamsson et al., 1991) and may also inhibit protein degradation, which is an effect of potential importance during immobilization periods when there is a net protein loss (Ye et al., 2013). PRP-released GFs and cytokines can also bind to fibrin and to proteoglycans in the extracellular matrix, constituting a storage pool that can be secondarily released by metalloproteases (Magra and Maffulli, 2005; Nurden, 2011). Actually, the mechanism of action is yet more complex, because the tissue outcomes may depend on the balance between plasma and platelet proteins (de Mos et al., 2008). However, the life-span of GFs and cytokines is relatively short even if repeated PRP injections can favor a better and long lasting action of platelet-derived GFs. So, it is conceivable that their effects can progressively fade until complete exhaustion.

Therefore, the persistent efficacy in the long-term (6–12 months), more than on a direct stimulation, probably relies on the activation of resident tendon stem/progenitor cells (TSPCs), which have been recently identified in tendons tissue from different animal species (Bi et al., 2007; Tempfer et al., 2009; Rui et al., 2010; Zhang et al., 2011; Mienaltowski et al., 2013). Like stem cells present in adult tissues, TSPCs are believed to be the source of newly differentiated tenocytes, responsible for maintaining adequate tenocyte numbers in the tissue throughout life and replenishing them after injury. Compared to bone marrow-derived mesenchymal stem cells, TSPCs express high levels of Scleraxis (a tendon-enriched specific transcription factor) and tenomodulin (a marker of adult tenocytes) and are able to form tendon and enthesis-like tissues when implanted *in vivo*. Morphologically, TSPCs possess smaller cell bodies and larger nuclei than ordinary tenocytes and have a cobblestone-like morphology in confluent cell cultures, whereas tenocytes are highly elongated, a typical phenotype of fibroblast-like cells (Zhang et al., 2011). TSPCs also proliferate more quickly than tenocytes in culture, and when implanted *in vivo* exhibit the ability to regenerate tendon-like tissues (Bi et al., 2007).

The biochemical niche, where TSPCs are embedded, is of paramount relevance for their appropriate maintenance and function. The importance of tendon extracellular matrix in the maintenance of TSPC stemness is supported by a recent study showing that rabbit Tendon Derived Stem Cells cultured on decellularized tendon matrix proliferated at a higher rate and had better stemness properties than those cultured on plastic tissue culture surface (Zhang et al., 2011). Moreover, GFs, as well as physiological loading, may increase TSPC numbers, by “awakening” or reactivating these cells (Sun et al., 2015). In conclusion, in young patients, it is likely that PRP administration, associated to eccentric training exercises, may activate the resident stem-cells, assuring the prosecution of the healing mechanism for several months afterward.

In contrast with findings in young subjects, this study shows that the PRP injections promote positive response in the clinical parameters, although less evident, in aged people. In the present research, the elderly subjects at baseline had similar VISA-A scores, symptoms duration and US degeneration. However, whereas in the short term (after 1–3 months), they showed an increase of VISA-A scores, although less relevant than in young subjects, in the medium and long-term (6–12 months) no further significant clinical improvement was observed. These results are not surprising, taking into account different factors.

First, aging is associated with a decline of plasma levels of Insulin-like GF-1 (Rudman et al., 1981; Zadik et al., 1985; Leifke et al., 2000; Moller et al., 2009; Boesen et al., 2014), and platelets of aged people release minor amounts of GFs. In this respect, Cho et al. (2011) and Lohmann et al. (2012) found that mesenchymal stem cells’ proliferation was higher with PRP from young donors, and that mesenchymal stem cells cultured with PRP from elder donors presented a senescent phenotype (Lohmann et al., 2012). Second, and most importantly, advanced age is associated to a numerical and/or functional deficits in resident populations of tenocytes and/or TSPCs. Senescent tenocytes become longer and thinner and have decreased protein synthesis, producing collagen fibers more disoriented with more variations in thickness. They also show a decrease in mucopolysaccharides, glycoaminoglycan, chondroitin sulfate, dermatan sulfate and in water content (Ippolito et al., 1980; Riley et al., 1994; Arnesen and Lawson, 2006; Thorpe et al., 2010; Ruzzini et al., 2014). Moreover, senescent cells are characterized by the elevated expression of senescent cell markers (β-gal), senescence-associated genes (p53, p21, and p16INK4a, metalloproteases, ADAMTS), and pro-inflammatory cytokines (Dimri et al., 1995; Campisi and d’Adda di Fagnana, 2007; Russo et al., 2015). Age-related changes in tenocyte behavior can be also responsible for altered migration and proliferation rate. Tsai et al. (2011) in an *in vitro* experiment performed on tenocytes derived from young, middle-age and old Sprague–Dawley rats, showed that decline in proliferation is directly correlated to aging and that aged tenocytes tend to stop in G0/G1 cellular phase. These results have been confirmed by several authors (Thorpe et al., 2010; Klatte-Schultz et al., 2012; Kostrominova and Brooks, 2013; Torricelli et al., 2013). Aged TSPC show similar characteristics, namely a profound TSPC self-renewal deficit accompanied with premature entry into cellular senescence; significant changes in the expression of genes regulating cell adhesion, migration, cytoskeleton (scleraxis and tenomodulin), dysregulated cell-matrix interactions and actin dynamics have been also observed (Kohler et al., 2013; McCharty and Hannafin, 2014). Interestingly, Zhou et al. (2010) showed that aged TSPCs formed adipocytes more readily than younger cells and expressed higher levels of adipogenic markers (PPARγ2, C/EBPa, and leptin) following induction. These data may help to explain the higher levels of adipose tissue normally associated with older tendons (Kannus and Jozsa, 1991), a pattern similar to that observed in bone marrow, where adiposity was found to correlate inversely with the functionality of hematopoietic stem/progenitor cells (Naveiras et al., 2009).

Therefore, a large number of concordant and sound biological data may explain why in aged persons PRP preparations can be less effective in promoting the activation of tenocytes and their progenitors cells, and therefore long-term tissue healing.

The findings of the present study can be hardly compared to those found in literature. Indeed, only few authors evaluated the impact of age on the therapeutic response to PRP (Ferrero et al., 2012; Boesen et al., 2014; Filardo et al., 2014). In these studies age resulted not influent on the outcomes, but the mean age of the patients was significantly lower, and aged persons were under-represented in the samples.

Some limitations of our research must be acknowledged. First, as well as for all the studies on PRP therapeutic activity, a key aspect to consider is the composition of the product used, because it cannot be excluded that other PRP formulations, different for cell type content, platelet concentration, storage modalities, activation methods, and protocol for therapeutic applications, could be more beneficial in aged people (Abate et al., 2012; Andia and Abate, 2012). Second, the data were retrospectively collected. However, in this respect, it must be observed that the patient’s selection was very careful and only those who had specific inclusion/exclusion criteria and were followed with a fixed experimental protocol were evaluated. Third, activity scales (i.e., Tegner Scale) was not collected; at this regard, it must be noted that sedentary lifestyle, which is more common in elderly subjects, may negatively influence the main outcomes of the study. In the future, a prospective trial, using PRP with higher platelet concentration, or with the addition of exogenous GFs, and enrolling a consistent number of subjects, will allow more sound and definitive conclusions.

### Ethical Statement

All procedures performed in study involving human participants were in accordance with the ethical standards of the institutional and/or national committee and with the 1964 Helsinki Declaration and its later amendments or comparable ethical standards. Informed consent was obtained from all participants included in the study.

## Author Contributions

VS: Design of the work; critical revision of the paper; final approval of the version to be published; agreement to be accountable for all aspects of the work in ensuring that questions related to the accuracy or integrity of any part of the work are appropriately investigated and resolved.

DV: Paper revision; final approval of the version; agreement to be accountable for all aspects of the work in ensuring that questions related to the accuracy or integrity of any part of the work are appropriately investigated and resolved.

AP: Paper revision; final approval of the version; agreement to be accountable for all aspects of the work in ensuring that questions related to the accuracy or integrity of any part of the work are appropriately investigated and resolved.

MA: Design of the work, acquisition, analysis, and interpretation of data; drafting and critical revision of the work; final approval of the version to be published; agreement to be accountable for all aspects of the work in ensuring that questions related to the accuracy or integrity of any part of the work are appropriately investigated and resolved.

## Conflict of Interest Statement

The authors declare that the research was conducted in the absence of any commercial or financial relationships that could be construed as a potential conflict of interest.
